# Links of Autoimmune Thyroid Disorders to Alzheimer’s Disease for Medicare Beneficiaries Ages 65+

**DOI:** 10.1093/geroni/igab046.1181

**Published:** 2021-12-17

**Authors:** Stanislav Kolpakov Nikitin, Arseniy Yashkin, Igor Akushevich

**Affiliations:** 1 Duke University, Durham, North Carolina, United States; 2 Duke University, Morrisville, North Carolina, United States

## Abstract

To explore racial disparities in the interaction between Hashimoto thyroiditis (HT), Graves disease (GD), and Alzheimer’s disease (AD)we used age-adjusted rates for Medicare beneficiaries aged 65+. We investigated race/ethnicity/gender-specific trends of age-adjusted prevalence of HT and GD over 1991-2017 and used the Cox proportional hazards model with propensity score matching to explore the associations between AD and these thyroid disorders. The total age-adjusted prevalence of GD was increasing over the study period reaching a maximum of 0.35% in 2014 followed by a gradual decline. Except for relatively high prevalence rates in Native American Males, no statistically significant sex/race-related differences were identified. GD was found to be associated with an increased risk of AD onset [HR: 1.129; CI: 1.050-1.215]. The mechanism of interaction between thyroiditis and AD could follow several alternative pathways. The primary mechanism involves changes in blood vessel function, mostly arterial wall stiffness, which leads to increased pulse wave velocity and consequently to the higher amplitude of oscillation of the peripheral microcapillary system in the brain leading to cerebral leukoaraiosis and damage in kidney tissue. This, in turn, leads to kidney diseases (themselves associated with AD/ADRD), increased for HT and decreased for GD LDL cholesterol levels and an increased for HT and decreased for GD total/HDL cholesterol ratio, which has an important role in increased common carotid intima‐media thickness (CIMT) in hypothyroid patients and is linked to arteriosclerosis. Additionally, both, endothelial dysfunction and arterial stiffness are risk factors for coronary or artery disease and consequently increase risks for AD/ADRD.

